# Multiplex LAMP Detection of the Genus *Phytophthora* and Four *Phytophthora* Species *P. ramorum*, *P. lateralis*, *P. kernoviae*, and *P. nicotianae*, with a Plant Internal Control

**DOI:** 10.1264/jsme2.ME21019

**Published:** 2021-06-10

**Authors:** Ayaka Hieno, Mingzhu Li, Kayoko Otsubo, Haruhisa Suga, Koji Kageyama

**Affiliations:** 1 River Basin Research Center, Gifu University, 1–1 Yanagido, Gifu city, Gifu 501–1193, Japan; 2 College of Life Sciences, Shaanxi Normal University, Xi’an, China; 3 Life Science Research Center, Gifu University, 1–1 Yanagido, Gifu city, Gifu 501–1193, Japan

**Keywords:** loop-mediated isothermal amplification, internal control, *Phytophthora ramorum*, *Phytophthora lateralis*, *Phytophthora kernoviae*

## Abstract

*Phytophthora* species cause destructive plant diseases worldwide. All *Phytophthora* species, except for one, are listed as plant quarantine organisms in Japan. The exception, *Phytophthora nicotianae* is considered to be a domestic species. The injurious pests *Phytophthora ramorum*, *Phytophthora lateralis*, and *Phytophthora kernoviae* are invasive pathogens that cause tree mortality worldwide, mainly in the United States and the United Kingdom. To effectively control *Phytophthora* diseases, we established detection methods that utilize the loop-mediated isothermal amplification (LAMP) of the genus *Phytophthora* and the four species *P. ramorum*, *P. lateralis*, *P. kernoviae*, and *P. nicotianae*. LAMP primers for *P. ramorum*, *P. lateralis*, and *P. kernoviae* were newly designed in the present study. Our multiplex assay includes the detection of plant DNA as an internal control. When the optimum ratio between plant and pathogen primers was used in multiplex LAMP assays, 1 pg to 100 fg of pathogen DNA was detected with similar sensitivity to that in simplex LAMP assays. The detection of plant DNA in the absence of pathogens enables us to check for and avoid undesirable negative results caused by enzyme inactivation or the contamination of amplification inhibitors from plant tissues. The total time from sample collection to results is approximately 120‍ ‍min, and, thus, our multiplex LAMP assay may be used as an accurate and time-saving detection method for *Phytophthora* pathogens.

Several *Phytophthora* species cause destructive diseases in forest trees and nursery plants (Lévesque, 2011; Roy *et al.*, 2014; Jung *et al.*, 2018). Three invasive species, *Phytophthora ramorum*, *Phytophthora lateralis*, and *Phytophthora kernoviae* cause widespread tree mortality and are potential threats to forests globally (Brasier *et al.*, 2005; Rizzo *et al.*, 2005; Webber, 2009; Robin *et al.*, 2011). *Phytophthora ramorum* Werres, de Cock & Man in ’t Veld is a causal agent of sudden oak death. It causes bleeding cankers and foliar lesions, which lead to the decline of the tree (Werres *et al.*, 2001). This disease was initially recognized in 1994–1995 and has attacked forest ecosystems in North America (Rizzo and Garbelotto, 2003) and Europe (Brasier *et al.*, 2004). *P. ramorum* has been reported from at least 109 plant species in 51 genera and 25 families (as of 2019; USDA), and has been detected in 30 countries (Invasive Species Compendium, last updated on 28 Jan 2021). The various host plants of *P. ramorum* are divided into two groups based on the risk of secondary infection; they are either transmissive (sporulating) or dead-end (non-sporulating) hosts. In forests in California and Oregon in the United States (US), *P. ramorum* causes trunk cankers on susceptible oak species (*Quercus* spp.) without sporulation, but does not cause leaf or twig symptoms (Rizzo *et al.*, 2005). On the other hand, bay laurel (*Umbellularia californica*) infected with *P. ramorum* shows leaf blight followed by the appearance of abundant sporangia on the leaves (Davidson *et al.*, 2002, 2005). Furthermore, when *P. ramorum* infected tanoak (*Lithocarpus densiflorus*) in North America (Rizzo *et al.*, 2002, 2005) and also Japanese larch (*Larix kaempferi*) in the United Kingdom (UK) (Webber *et al.*, 2010) sporangia appeared on the foliage and were the source of inocula dispersed by rain. In global terms, the pathogen may spread rapidly and widely due to the growing demand for ornamental trees (Brasier *et al.*, 2004; Brasier and Webber, 2010). Tree species of the genera *Camellia*, *Pieris*, *Rhododendron*, *Syringa*, and *Viburnum* are considered to be the key hosts involved in moving this pathogen to new geographical areas (Grünwald *et al.*, 2008).

*P. ramorum* was initially detected in the UK in 2002 (Lane *et al.*, 2003), and affected locations were surveyed to assess the extent of pathogen occurrence (Brasier *et al.*, 2005). During these surveys, another invasive pathogen, *P. kernoviae* Brasier, Beales & S. A. Kirk, was isolated in 2003 (Brasier *et al.*, 2005). *P. kernoviae* forms caduceus sporangia on plant foliage, suggesting its aerial or splash dispersal (Brasier *et al.*, 2005). Some *Rhododendron* plants in the UK were previously found to be infected by both *P. ramorum* and *P. kernoviae* (Webber, 2009). Abundant sporangia are produced on the leaves of infected rhododendrons, and this host has played a key role in the spread of *P. ramorum* and *P. kernoviae* in the natural environment (Webber, 2009). *P. kernoviae* has a wide host range, infecting at least 43 plant species in 21 genera and 14 families (USDA Agricultural Research Service). In addition to the UK (Brasier *et al.*, 2005), *P. kernoviae* has been found in New Zealand (Scott and Williams, 2014) and recently in Chile (Sanfuentes *et al.*, 2016).

*P. lateralis* Tucker & Milbrath is another tree pathogen that mainly infects Cupressaceae plants. In North America, it has killed Port Orford cedar (*Chamaecyparis lawsoniana*) and occasionally Pacific yew (*Taxus brevifolia*) (Hansen *et al.*, 2000). *P. lateralis* was initially detected in North America in the 1920s (Hansen *et al.*, 2000), and by the 1950s, had spread throughout the native range of Port Orford cedar in southern Oregon and northern California (Brasier *et al.*, 2010). In 2009, a major outbreak of Port Orford cedar mortality caused by *P. lateralis* was reported in France (Robin *et al.*, 2011). *P. lateralis* produces abundant chlamydospores that enable it to survive in dry and hot environments (Jung *et al.*, 2018). This species also produces caduceus sporangia, suggesting its aerial dispersal (Robin *et al.*, 2011).

In the quarantine control of *Phytophthora* species, such as *P. kernoviae*, symptomless infections are assumed even if a phytosanitary certificate is attached (Fichtner *et al.*, 2012). Therefore, imported plants must be carefully inspected for the presence of these pathogens, particularly in the case of transmissive host plants. Various effective detection methods have been developed, such as recombinase polymerase amplification-based methods (Miles *et al.*, 2015) and TaqMan-based real-time PCR methods (Schena *et al.*, 2006; Feau *et al.*, 2019). However, high throughput sampling is technically difficult because DNA extraction may be time-consuming. Therefore, we developed a loop-mediated isothermal amplification (LAMP)-based method (Notomi *et al.*, 2000; Mori *et al.*, 2001; Nagamine *et al.*, 2002; Mori and Notomi, 2009). LAMP has the advantages of high tolerance to amplification inhibitors (Kaneko *et al.*, 2007) and, thus, simple and easy DNA extraction methods are applicable for LAMP-based detection.

LAMP detection multiplexed with an internal control is useful for the accurate detection of plant pathogens. The internal control shows whether the amplification was successful, thereby allowing us to check for and avoid undesirable negative results caused by enzyme inactivation or amplification inhibitors from plant tissues. We used a previously published plant primer set for this internal control (Tomlinson *et al.*, 2010), and made modifications to expand the number of detectable plant families. Regarding pathogen detection, we used primer sets for the detection of all species in the genus *Phytophthora*, and four species-specific primer sets for the detection of *P. ramorum*, *P. lateralis*, *P. kernoviae*, and *P. nicotianae*. We designed new primer sets for the three pathogens *P. ramorum*, *P. lateralis*, and *P. kernoviae*. Primers for *P. ramorum* (Tomlinson *et al.*, 2007) and *P. kernoviae* (Tomlinson *et al.*, 2010) have previously been reported; however, we designed new primers that were a better fit for our experimental conditions (different enzyme and reaction buffer), reduced undesirable amplification, and increased the specificity of detection. This was necessary due to the high levels of sequence similarity among the target and non-target species in the primer-tagged DNA region. The non-target species *Phytophthora morindae* Z. G. Abad & S. C. Nelson (Nelson and Abad, 2010) is phylogenetically closely related to the target species *P. kernoviae*. In Japan, all species in the genus *Phytophthora*, except for one, are listed as plant quarantine organisms. The exception is *P. nicotianae*, which is considered to be a domestic species. In our LAMP-based detection method, we included the genus-specific primer set for *Phytophthora* (Hieno *et al.*, 2020); therefore, we may identify plant materials infected with any *Phytophthora* species, and the species-specific primer set for *P. nicotianae* (Hieno *et al.*, 2019) to identify plants infected only with *P. nicotianae*. Our multiplex LAMP-based detection method with the internal plant control may be used for the effective quarantine control of *Phytophthora* pathogens in plants.

## Materials and Methods

### Isolates and mycelial DNA extraction

The isolates used in the present study are listed in Table S1. They were grown on V8 agar plates at 25°C (V8 agar [Miller, 1955] was used with the following modifications: 162‍ ‍mL V8 juice [Campbell Japan] was mixed with 2.5‍ ‍g CaCO_3_ for 30‍ ‍min, then transferred to a 50-mL tube and centrifuged at 4,000‍ ‍rpm for 10‍ ‍min. The supernatant was collected and diluted with deionized water to a total volume of 1 L. Agar [2% {w/v}] was added, and the mixture was autoclaved. The resulting V8 agar medium was plated on 6-cm plastic Petri dishes [5‍ ‍mL per plate]). DNA were extracted from mycelial colonies using the PrepMan^TM^ Ultra Sample Preparation Reagent (Thermo Fisher Scientific). A small amount of the mycelial mat was collected by scraping with an inoculating needle, transferred to a 1.5-mL Eppendorf tube containing 100‍ ‍μL of 50% PrepMan Ultra Reagent, and incubated at 100°C for 10‍ ‍min. After a 3-min incubation at room temperature, the sample was centrifuged at 21,880×*g* for 3‍ ‍min. The supernatant (approximately 80‍ ‍μL) was transferred to a new 1.5-mL tube and the total DNA concentration was measured using the QuantiFluor^®^ dsDNA System (Promega) with a Qubit^®^2.0 Fluorometer (Invitrogen). The concentration was adjusted to 100 pg μL^–1^ with sterilized deionized water (SDW) and stored at 4°C until used.

### LAMP primer design

The sequences of the LAMP primer sets are shown in Table 1. The accession numbers of sequences used for primer design are shown in Fig. S1, S2, S3, and S4. The primer sets for *P. ramorum*, *P. lateralis*, and *P. kernoviae* were designed using Primer Explorer V5 software (Eiken Chemical, https://primerexplorer.jp). The primers for *P. ramorum* and *P. lateralis* were based on the cytochrome *c* oxidase subunit I (*cox*1) gene of the isolates CBS 101553 (from the CBS-KNAW collection, Westerdijk Fungal Biodiversity Institute, the Netherlands) and CPHST BL 42 (from the USDA-APHIS Center for Plant Health Science and Technology, Beltsville Laboratory, the United States), respectively (Fig. S1 and S2). The primers for *P. kernoviae* were based on the rDNA-ITS region of the isolate P1571 (Fig. S3). Each LAMP primer set included the F3, B3, FIP, and BIP primers, but not the F- or B-Loop primer (Table 1 and Fig. S1, S2, and S3).

The plant LAMP primer set previously reported (Tomlinson *et al.*, 2010) was modified based on multiple alignments in the *cox*1 gene (Fig. S4 and Table S2) and additional primers were designed to expand the number of detectable plant families. The plant LAMP primer set consisted of 8 primers: F3, B3G, FIPA (F1cA to F2), FIP2 (F1c-2 to F2-2), BIPA (B1cA to B2), F-Loop, F-Loop2, and B-loop (Table 1 and Fig. S4).

### Extraction of plant DNA

The plant samples used in the present study are listed in Table 2 and Table S3. DNA samples were extracted from inoculated and non-inoculated plants using Kaneka Easy DNA Extraction Kit version 2 (Kaneka). Regarding thin plant tissue samples, such as leaves, buds, and flower buds, a 5-mm square of tissue was collected in a 2-mL screw-cap tube (BIO-BIK, INA OPTIKA) containing a stainless steel bead (0.25 inches, SUS304; AS ONE). Regarding stem and rhizome samples, the tissue was shaved off with a knife and two pieces (lengths of 5‍ ‍mm) were collected in a tube as described above. Solution A of the Kaneka kit (200‍ ‍μL) was added and the tube was vortexed using Vortex-Genie2 with a Turbomix attachment (Scientific Industries) for 5‍ ‍min. The tube was briefly centrifuged to collect all drops at the bottom and then heated at 98°C for 8‍ ‍min in a heat block. After cooling to room temperature, 28‍ ‍μL of Solution B was added, mixed well, and then centrifuged at 2,000×*g* for 3‍ ‍min in a micro-centrifuge (XX42CF0RT, CHIBITAN-R; Merck Millipore). The supernatant was transferred to a 1.5-mL Eppendorf tube and centrifuged under the same conditions. The supernatant was transferred to a new 1.5-mL tube and diluted 20 times with SDW. Samples were stored at 4°C until used as templates.

When weak or no detection results were obtained, extracted DNA was subjected to additional purification using the OneStep^TM^ PCR Inhibitor Removal Kit (Zymo Research). The non-diluted DNA extract (50 or 100‍ ‍μL) was purified according to the manufacturer’s protocol. Samples were stored at 4°C until used as templates.

### Pathogen inoculation

The inoculated host plants and *Phytophthora* isolates used in the present study are listed in Table 2. Tomato and eggplant fruits inoculated with *P. nicotianae* and *P. capsici* were prepared as previously described (Hieno *et al.*, 2020). Since *P. nicotianae* and *P. capsici* infect the same host, *P. capsici* was used in this experiment to confirm the species-specific detection of *P. nicotianae*.

The attached and detached tree leaves of rhododendron (*Rhododendron* sp.), Japanese andromeda (*Pieris japonica* ssp. *japonica*), and common camellia (*Camellia japonica*) were also used in this experiment. Isolates of *P. ramorum*, *P. lateralis*, *P. kernoviae*, *Phytophthora foliorum*, and *Phytophthora hibernalis* were used to inoculate the leaves. *P. foliorum* and *P. hibernalis* are phylogenetically closely related to *P. ramorum* and *P. lateralis*; all four species belong to subclade 8c (Abad *et al.*, 2019). Therefore, *P. foliorum* and *P. hibernalis* were used to confirm the species-specific detection of *P. ramorum* and *P. lateralis*. These isolates were grown on V8 agar plates for 4‍ ‍d, and 7-mm mycelial disks were taken from actively growing colonies. Detached leaves with their petioles wrapped in wet paper were placed on lattices in plastic tube boxes with a wet paper towel at the bottom, and each was inoculated with one or two of the mycelial disks. The boxes were lidded to maintain high humidity. Leaves on trees were inoculated with mycelial disks and wrapped in parafilm, and each entire leaf was then covered with a plastic Ziploc bag. The whole plant was placed in a large plastic case to maintain high humidity. After the inoculation, leaves and plants were incubated in a growth chamber with a cycle of 12 h, 20°C, light and 12 h, 15°C, dark for 3–5 d. After the incubation, 5-mm squares of infected tissue were examined for DNA extraction as described above.

### LAMP assays

The LAMP reaction was conducted in a total volume of 25‍ ‍μL containing 1× Isothermal Master Mix (fluorescent dye; Optigene), 4‍ ‍mg mL^–1^ bovine serum albumin (BSA, fraction V; Sigma), primer mixture(s), and a DNA template: 1‍ ‍μL (default) or 2‍ ‍μL (for DNA extracted from plants). The 1× primer mixture for each pathogen contained 0.2‍ ‍μM each of the F3 and B3 primers (0.05‍ ‍μM for *P. nicotianae*) and 1.6‍ ‍μM each of the FIP and BIP primers. The 1× mixture for the previously designed plant LAMP primers (Tomlinson *et al.*, 2010) contained 0.2‍ ‍μM each of the F3 and B3 primers, 1.6‍ ‍μM each of the FIP and BIP primers, and 0.8‍ ‍μM each of the F-Loop and B-loop primers. The 1× mixture for the plant LAMP primer set modified in the present study contained 0.2‍ ‍μM each of the F3 and B3G primers, 0.8‍ ‍μM each of the FIPA and FIP2 primers, 1.6‍ ‍μM of the BIPA primer, 0.4‍ ‍μM each of the F-Loop and F-Loop2 primers, and 0.8‍ ‍μM of the B-loop primer. In simplex LAMP, the primer mixtures for the pathogens were used at 1× concentration and the previously designed or modified plant primer mixture were used at 0.08× concentration. In multiplex LAMP, the primer mixtures for the pathogens were used at 1× concentration. The modified plant primer mixture was tested at 0.2×, 0.15×, 0.1×, 0.08×, and 0.05× concentrations. A reaction mix containing SDW instead of DNA was used as a negative control. The LAMP assay was performed on the portable real-time fluorometer Genie^®^II (Optigene) with the following conditions: pre-heat at 65 or 68°C for 5‍ ‍min; amplification at 65 or 68°C for 60‍ ‍min; and an annealing curve analysis at 98 to 80°C, ramping at 0.05°C s^–1^. The temperatures of the pre-heat and amplification steps were optimized at 65°C for all primer sets, except *Phytophthora* genus-specific primers, which were optimized at 68°C. Therefore, multiplex assays were performed at 65°C for all assays, except those that included the *Phytophthora* genus-specific primers, which were performed at 68°C. The format of the raw data (.gen) was changed to a text file using Genie Explorer version 2.0.6.3 software (Optigene), and amplification curves and annealing curves were then created using Microsoft Excel. The sensitivity of each LAMP assay was assessed with at least three experiments. The specificity and detectability of each assay was evaluated at least twice for each DNA sample.

## Results

### Primer design and specificity tests of species-specific LAMP primer sets

Regarding the species-specific detection of *P. ramorum*, *P. lateralis*, and *P. kernoviae*, we designed LAMP primer sets (Table 1) based on multiple alignments of the *cox*1 gene for *P. ramorum* and *P. lateralis* (Fig. S1 and S2), and the rDNA-ITS sequence for *P. kernoviae* (Fig. S3). The specificity of each primer set was tested using mycelial DNA, which was extracted from *Phytophthora* and closely related genera. There were 61 taxa including subspecies (101 isolates) of *Phytophthora*, 12 species (12 isolates) of *Pythium*, one species of *Phytopythium*, and one isolate each of the following soil-borne pathogens: *Aphanomyces cochlioides*, *Fusarium oxysporum* f. sp. *fragariae*, *Plasmodiophora brassicae*, *Rhizoctonia solani*, *Saprolegnia parasitica*, *Sclerotinia sclerotiorum*, and *Verticillium albo-atrum* (Table S1). The results shown in Table S1 indicated that the primer sets were specific for each of the three species.

### Primer modification and evaluation of the detection range of the plant LAMP primer set

We modified a previously designed plant LAMP primer set (Tomlinson *et al.*, 2010) to expand the number of detectable plant families. Based on multiple alignments of the *cox*1 gene (Fig. S4 and Table S2), primer sequences were modified and additional primers were designed (Table 1). The modified plant LAMP primer set was tested with DNA extracted from 176 plant species representing 155 genera and 110 families (Table S3). The reaction was performed using a 0.08× primer mixture at 65°C for 60‍ ‍min; this concentration was selected as the optimum for species-specific multiplex LAMP reactions at 65°C. Under these conditions, we detected 140 plant species of 124 genera belonging to 87 families, including 10 species of 8 genera belonging to 5 families that were not amplified using the previously designed primers (Tomlinson *et al.*, 2010) (Table S3). Among the weakly and non-detected species, additional DNA purification improved detectability with the modified primer set for 12 plant species of 12 genera belonging to 12 families (Table S3).

### Sensitivity of species-specific LAMP primer sets

To test the detection limits of the LAMP primers for *P. ramorum*, *P. lateralis*, and *P. kernoviae*, we used mycelial DNA extracted from each species, with amounts ranging between 1 fg and 100 pg. *P. ramorum* PR-06-021, Pr-1, and CBS 101553; *P. lateralis* WPC P3361 (from the World Phytophthora Genetic Resource Collection, the United States), 450, and ATCC 201856 (from the American Type Culture Collection, the United States); and *P. kernoviae* Pk-1, 2654, and P1571 were tested. The specificity of each amplification was confirmed with a single peak in the annealing curve analysis at approximately 82°C for *P. ramorum* (Fig. S5D), 83°C for *P. lateralis* (Fig. S5E), and 86°C for *P. kernoviae* (Fig. S5F). The detection limits for *P. ramorum* (isolates PR-06-021 and Pr-1), *P. lateralis* (isolates WPC P3361 and ATCC 201856), and *P. kernoviae* (isolates Pk-1 and P1571) were 10, 100, and 10 fg, respectively. Representative data are shown in Fig. S5. On the other hand, we found 10-fold lower sensitivities for *P. ramorum* isolate CBS 101553, *P. lateralis* isolate 450, and *P. kernoviae* isolate 2654 (data not shown).

### Selection of optimum primer ratios for multiplex LAMP assays

The optimum primer ratios for multiplex LAMP assays designed to detect pathogens in plant samples were selected. “Optimum” indicates the concentration of plant primers that may be used without disturbing pathogen detection, while functioning as an internal control for non-infected plants. We used the 1× concentration of the pathogen primer mixes combined with different concentrations of the plant primer mix, and tested these mixtures using reaction temperatures of 65 or 68°C in the pre-heat and amplification steps.

**Fig. 1.**
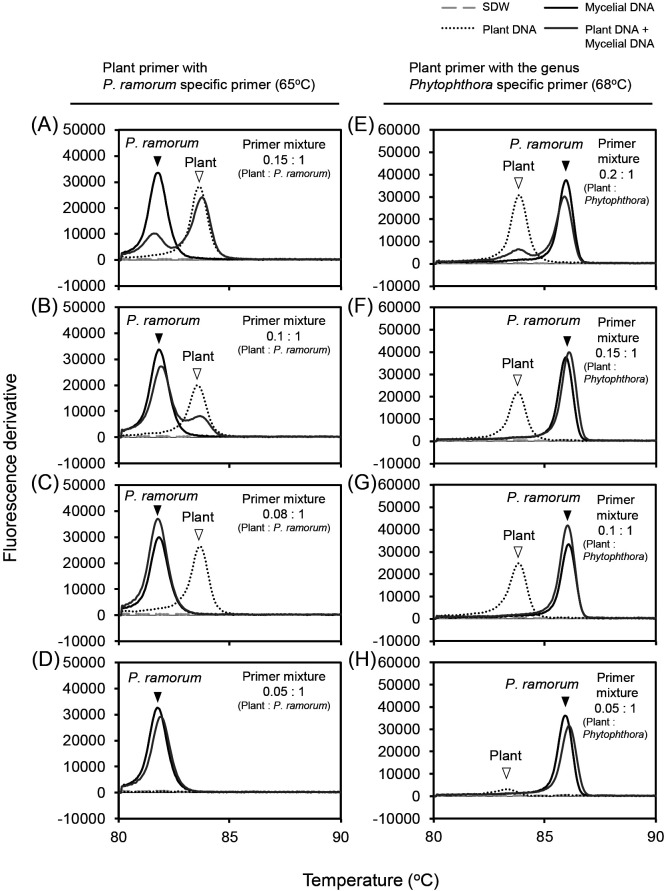
Selection of optimum ratios between plant primers and *Phytophthora* species- or genus-specific primers for multiplex LAMP assays. (A, B, C, and D) Primers for *P. ramorum* were mixed with different amounts of the plant primers. (E, F, G, and H) *Phytophthora* genus-specific primers were mixed with different amounts of the plant primers. Four DNA mixes were tested with each primer mixture: 1) SDW as a negative control, 2) plant DNA extracted from a *Rhododendron* sp. (71.5‍ ‍ng) only, 3) *P. ramorum* DNA (1 pg) only, and 4) a mixture of plant DNA (71.5‍ ‍ng) and *P. ramorum* DNA (1 pg). After amplification at 65°C (A, B, C, and D) or 68°C (E, F, G, and H) for 60‍ ‍min, fluorescence derivative data during the annealing phase (98 to 80°C) were obtained. Open and black arrowheads indicate peaks derived from plant DNA and *P. ramorum* DNA, respectively. SDW: sterilized deionized water.

To select the optimum ratio in the species-specific multiplex LAMP detection of *P. ramorum*, *P. lateralis*, *P. kernoviae*, and *P. nicotianae*, we used the *P. ramorum* primers in reactions performed at 65°C. The *P. ramorum* species-specific primer set (at the 1× concentration) was mixed with different concentrations of the modified plant primer set (0.15×, 0.1×, 0.08×, and 0.05×), and then tested in assays with mycelial DNA extracted from *P. ramorum* Pr-1 and/or plant DNA extracted from the leaves of *Rhododendron* sp. (Fig. 1). Four samples were prepared and used for these multiplex LAMP assays: 1) SDW as a negative control, 2) plant DNA only (71.5‍ ‍ng), 3) pathogen (*P. ramorum*) DNA only (1 pg), and 4) a mixture of plant DNA (71.5‍ ‍ng) and pathogen DNA (1 pg). In reactions containing the 0.15× or 0.1× plant primers with both types of DNA, pathogen DNA (peak at approximately 82°C) and plant DNA (peak at approximately 84°C) were both simultaneously detected (Fig. 1A and B). In these cases, the peaks from pathogen DNA were lower than those when only pathogen DNA was included in the reaction. This suggested that pathogen detection was disturbed by competitive amplification with plant DNA. In reactions with the 0.08× or 0.05× plant primer and both types of DNA, only pathogen DNA was detected (Fig. 1C and D). In the reaction with the 0.08× plant primer and both DNAs, the pathogen peak was higher than that when only pathogen DNA was included (Fig. 1C), implying that detection of the pathogen was not disturbed. The 0.05× plant primer mix was not able to detect plant DNA (missing peak at approximately 84°C) in any of the reactions (Fig. 1D). Based on these results, the optimum primer ratio for the reaction temperature of 65°C was 1× *P. ramorum* species-specific primers with 0.08× plant primers. With this ratio, there was no significant interference with pathogen detection, and plant DNA was still detected in the absence of pathogen DNA. This ratio was also optimum with the other pathogen primers (data not shown).

In multiplex LAMP assays designed to specifically detect all *Phytophthora* species, the amplification reaction was performed at 68°C. We used *Phytophthora* genus-specific primers (1×) combined with different concentrations of the modified plant primers (0.2×, 0.15×, 0.1×, and 0.05×) and assayed with the DNA samples described above. In reactions containing the 0.2× plant primer and both types of DNA, pathogen DNA (peak at approximately 86°C) and plant DNA (peak at approximately 84°C) were simultaneously detected (Fig. 1E). The pathogen peak was lower than that when plant DNA was absent. This suggested that the amplification of pathogen DNA was under competition. When the reaction contained the 0.15×, 0.1×, or 0.05× plant primer and both types of DNA, only pathogen DNA was detected (Fig. 1F, G, and H). Furthermore, the 0.05× plant primer gave the very weak detection of plant DNA in the reaction containing only plant DNA (Fig. 1H). Based on these results, the optimum primer ratio in reactions at 68°C was 1× *Phytophthora* genus-specific primers with 0.15× plant primers because this ratio showed no evidence of interference in the amplification of pathogen DNA, but also allowed for the amplification of plant DNA when pathogen DNA was absent.

### Sensitivity of multiplex LAMP assays for the detection of pathogens

**Fig. 2.**
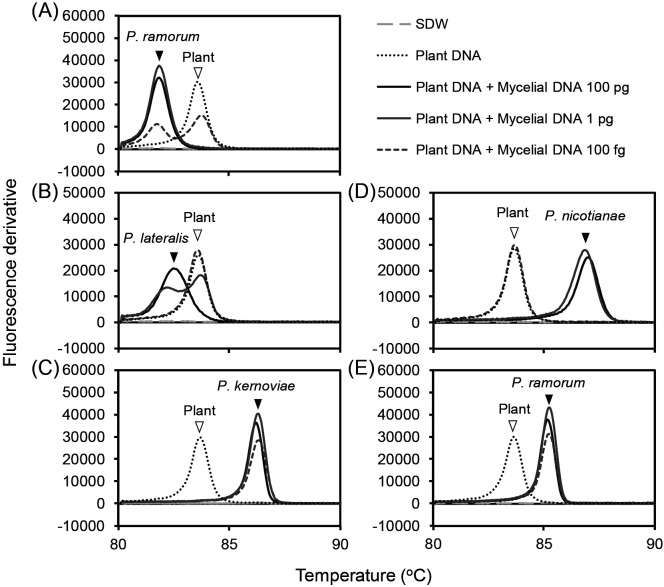
Sensitivity of the multiplex LAMP assay using the plant primer set and each *Phytophthora* species- or genus-specific primer set. Plant DNA extracted from a *Rhododendron* sp. (71.5‍ ‍ng per reaction) was mixed with serially diluted mycelial DNA (1 fg to 100 pg) from each *Phytophthora* species, and subjected to multiplex LAMP assays. Representative data for the 100 fg to 100 pg assays are shown. The plant primer set was mixed with each pathogen primer set and tested with the serially diluted DNA samples as follows: (A) *P. ramorum* primers and *P. ramorum* Pr-1 DNA, (B) *P. lateralis* primers and *P. lateralis* WPC P3361 DNA, (C) *P. kernoviae* primers and *P. kernoviae* P1571 DNA, (D) *P. nicotianae* primers and *P. nicotianae* CBS 305.29 DNA, and (E) *Phytophthora* genus-specific primers and *P. ramorum* Pr-1 DNA. Representative data are shown. Plant:pathogen primer ratios were 0.08:1 in (A, B, C, and D) and 0.15:1 in (E). After amplification at 65°C (A, B, C, and D) or 68°C (E) for 60‍ ‍min, fluorescence derivative data during the annealing phase (98 to 80°C) were obtained. Open and black arrowheads indicate peaks derived from plant DNA and *Phytophthora* species DNA, respectively. SDW: sterilized deionized water.

The pathogen detection limits of multiplex LAMP assays were tested using specific primer sets for the genus *Phytophthora* (Hieno *et al.*, 2020), *P. ramorum*, *P. lateralis*, and *P. kernoviae* (the present study), and *P. nicotianae* (Hieno *et al.*, 2019) multiplexed with modified plant primers (Tomlinson *et al.*, 2010). These primer sets were mixed at the optimized ratios described above. Mycelial DNA (ranging between 1 fg and 100 pg) and/or plant DNA (71.5‍ ‍ng) extracted from *Rhododendron* sp. leaves were used for the assay. Fig. 2 shows representative data for assays with 100 fg, 1 pg, and 100 pg mycelial DNA. Pathogen DNAs were detected as peaks at approximately 82°C for *P. ramorum* Pr-1 (Fig. 2A), 83°C for *P. lateralis* WPC P3361 (Fig. 2B), 86°C for *P. kernoviae* P1571 (Fig. 2C), and 87°C for *P. nicotianae* CBS 305.29 (Fig. 2D). When *Phytophthora* genus-specific primers were used to detect *P. ramorum* Pr-1 DNA, the peak occurred at approximately 85°C (Fig. 2E). Plant DNA was detected as a peak at approximately 84°C (Fig. 2A, B, C, D, and E). The detection limits were 100 fg in multiplex LAMP assays using the primer sets for *P. ramorum*, *P. kernoviae*, and the genus *Phytophthora* (Fig. 2A, C, and E), and 1 pg for assays with the primer sets for *P. lateralis* and *P. nicotianae* (Fig. 2B and D).

### Detection of pathogens in inoculated plants

**Fig. 3.**
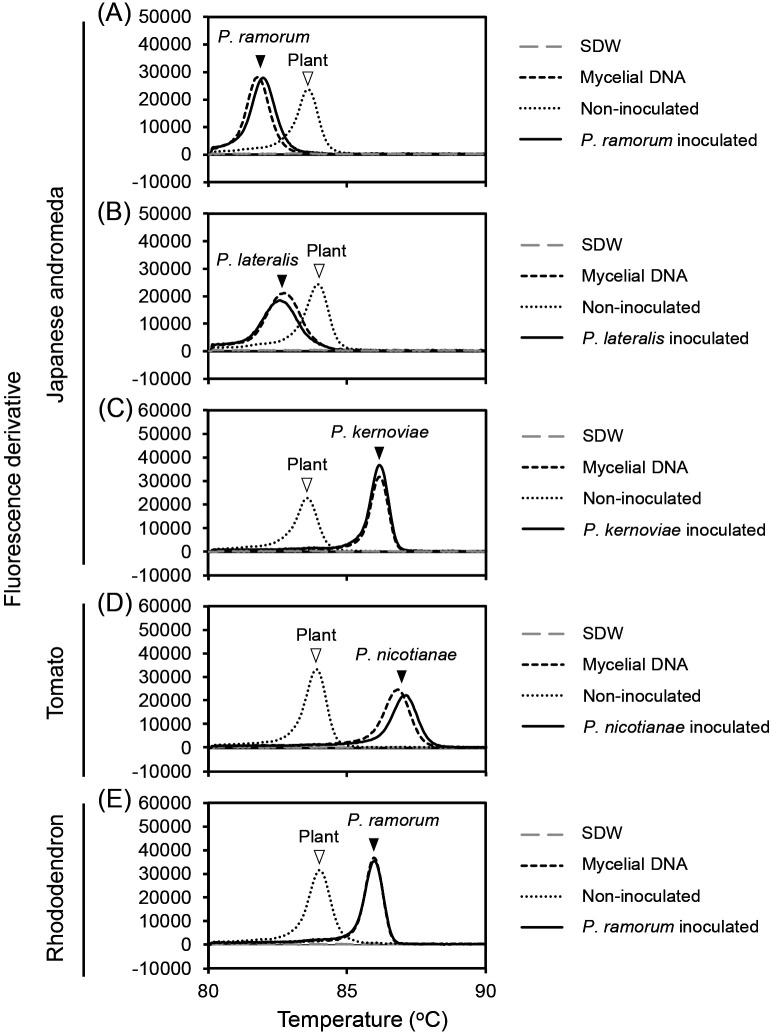
Detection of *Phytophthora* pathogens in inoculated plants using multiplex LAMP assays. Plant DNA extracted from inoculated plants and mycelial DNA (100 pg per reaction) extracted from each *Phytophthora* species were subjected to multiplex LAMP assays, with SDW and non-inoculated plants as controls. Plant primers were mixed with specific pathogen primers and tested with plant and mycelial DNA as follows: (A) *P. ramorum* primers, detached Japanese andromeda leaves inoculated with *P. ramorum* CBS 101330, and mycelial DNA from *P. ramorum* Pr-1; (B) *P. lateralis* primers, detached Japanese andromeda leaves inoculated with *P. lateralis* CBS 168.42, and mycelial DNA from *P. lateralis* WPC P3361; (C) *P. kernoviae* primers, detached Japanese andromeda leaves inoculated with *P. kernoviae* CBS 122051, and mycelial DNA from *P. kernoviae* P1571; (D) *P. nicotianae* primers, tomato fruits inoculated with *P. nicotianae* GK10Eg1, and mycelial DNA from *P. nicotianae* CBS 305.29; and (E) *Phytophthora* genus-specific primers, rhododendron leaves inoculated with *P. ramorum* CBS 101330, and mycelial DNA from *P. ramorum* Pr-1. Plant:pathogen primer ratios were 0.08:1 in (A, B, C, and D) and 0.15:1 in (E). After amplification at 65°C (A, B, C, and D) or 68°C (E) for 60‍ ‍min, fluorescence derivative data during the annealing phase (98 to 80°C) were obtained. Open and black arrowheads indicate peaks derived from plant DNA and *Phytophthora* species DNA, respectively. SDW: sterilized deionized water. See Table 2 for more results.

Multiplex LAMP assays for the detection of pathogens in inoculated plants were tested (Fig. 3 and Table 2). The attached and detached leaves of rhododendron (*Rhododendron* sp.) and Japanese andromeda (*Pieris japonica* ssp. *japonica*) were inoculated with *P. ramorum*, *P. lateralis*, *P. kernoviae*, *P. foliorum*, and *P. hibernalis*. The detached leaves of common camellia (*Camellia japonica*), which were inoculated with *P. ramorum*, *P. lateralis*, and *P. kernoviae*, were examined. Tomato (*Solanum lycopersicum*) and eggplant (*Solanum melongena*) fruits were inoculated with *P. capsici* and *P. nicotianae*. On days three to five post-inoculation, extracted DNA from the infected tissues were tested for the detection of the pathogen using multiplex LAMP assays. Target pathogen DNA with its specific primer set was detected as a single peak at the same temperatures as those described above for the sensitivity assays (see Fig. 2). Representative data are shown in Fig. 3. In control assays of samples from non-inoculated plants, plant DNA was amplified with a peak at approximately 84°C (Fig. 3). Therefore, multiplex LAMP assays detected infection by the target pathogen, and the modified plant primers functioned as an internal control.

## Discussion

To detect important pathogenic species of the genus *Phytophthora*, we designed species-specific LAMP primer sets for *P. ramorum*, *P. lateralis*, and *P. kernoviae* and tested their specificity with numerous *Phytophthora* species. Intra-species variation was present in the genome regions used for primer design. Therefore, we retrieved sequence data for *P. ramorum* (28 isolates), *P. lateralis* (42 isolates), and *P. kernoviae* (22 isolates) from the NCBI nucleotide database (https://www.ncbi.nlm.nih.gov/nucleotide/) to confirm the conservation of the species-specific sequences in the designed primers. In addition, the primer sets were tested with mycelial DNA from the isolates of *P. ramorum* (30 isolates), *P. lateralis* (4 isolates), and *P. kernoviae* (9 isolates) that had originated from different countries and/or host plants. All the isolates tested were detected by each species-specific primer. Therefore, intra-species sequence variations among the isolates examined did not affect our species-specific detection.

We modified a previously reported plant LAMP primer set (Tomlinson *et al.*, 2010) based on a multiple alignment analysis of plant DNA (Fig. S4). Additional FIP2 and F-loop2 primers were designed that annealed to plant DNA not amplified by the original primers. Additionally, the original primers contained mixed bases at some sites, and we selected the bases at these sites that were least likely to result in primer dimerization. The modified primer set (Table 1) was able to detect 140 plant species of 124 genera belonging to 89 families, and these included 10 plant species of 8 genera belonging to 5 families that were not detected with the unmodified primers (Table S3). The newly detectable species were mainly in the Pinales (Cupressaceae, Pinaceae, and Taxaceae). To the best of our knowledge, our plant LAMP primer set has the broadest detectability available. Plants containing saponin, such as *Pittosporum tobira*, were more difficult to detect (Vincken *et al.*, 2007; Itamiya and Kikkawa, 2016); however, additional DNA purification also improved their detection.

An internal control (the detection of plant DNA) is important when screening plant samples for infecting pathogens because it shows that negative results are not due to poor DNA extraction or other errors. The simultaneous amplification of the pathogen and plant DNA by multiplex LAMP is preferred, but is very difficult. In natural samples, the relative amounts of DNA originating from pathogens and plants markedly vary depending on the severity of the infection. In LAMP assays, the strong amplification of more abundant DNA may overwhelm the amplification of less abundant DNA (Kubota and Jenkins, 2015). Furthermore, the sensitivity of the assay may be reduced in multiplex reactions (Kubota and Jenkins, 2015; Yasuhara-Bell *et al.*, 2018). Based on these findings, it is very important to optimize the primer ratio. We found optimum ratios of 1× species-specific primers with 0.08× plant primers for assays at 65°C, and 1× genus-specific primers with 0.15× plant primers for assays at 68°C (Fig. 1). The concentrations of the pathogen primers were similar to those in simplex LAMP to maintain sensitivity, and the concentration of the plant primer set was markedly lower in order to prevent pathogen detection being impeded by the presence of plant primers. Under these optimum primer ratios, we detected the target DNA of each pathogen in multiplex assays with similar sensitivity to simplex assays (between 1 pg and 100 fg) even though plant DNA was present at a markedly larger amount (71.5‍ ‍ng) than pathogen DNA (Fig. 2). When we tested low concentrations of pathogen DNA (1 pg for *P. lateralis* and 100 fg for *P. ramorum*), the annealing peak of pathogen DNA was indistinct or formed dual peaks with plant DNA (Fig. 2). This result suggests that the amplification of pathogen DNA was slightly reduced under competition with plant DNA. Nevertheless, we selected the primer ratios indicated above because 0.08× was the lowest concentration of plant primers that clearly detected plant DNA (Fig. 1).

The genus-specific multiplex LAMP assay developed in the present study is applicable to various conditions in *Phytophthora* disease management. The genus *Phytophthora* has a large number of host plants. Based on database searches in 2019, mainly from the USDA Agricultural Research Service, approximately 2,600 host plant species of 956 genera belonging to 184 families have been reported to host *Phytophthora* species worldwide. Moreover, previous studies suggested that global warming enhances disease development and affects the distribution of *Phytophthora* pathogens (Brasier, 1996; Kaukoranta, 1996; Rafiei and Banihashemi, 2013; Kamoun *et al.*, 2015). This implies that disease control will become increasingly important. Specific fungicides are available for oomycetes, including metalaxyl-M, zoxamide, fluopicolide, cyazofamid, amisulbrom, ametoctradin, propamocarb, dimethomorph, iprovalicarb, mandipropamid, cymoxanil, Fosetyl-Al, and ethaboxam (Kuck *et al.*, 2011); benthiavalicarb-isopropyl (Miyake *et al.*, 2005); and oxathiapiprolin (Leadbeater, 2015; Wu *et al.*, 2019). Therefore, an accurate diagnosis is crucial. It is also important to diagnose *Phytophthora* disease in its early stages in order to control it, and molecular-based diagnosis methods are needed for this purpose.

In the present study, a multiplex LAMP detection method was developed for the genus *Phytophthora* and four species in the genus, *P. ramorum*, *P. lateralis*, and *P. kernoviae*, and *P. nicotianae*. Our multiplex assay includes the detection of plant DNA as an internal control. The detection of plant DNA in the absence of pathogens enables us to prevent undesirable negative results. The total time from sample collection to results is approximately 120‍ ‍min. Therefore, our multiplex LAMP assay may be used as an accurate and time-saving detection method for *Phytophthora* pathogens.

## Citation

Hieno, A., Li, M., Otsubo, K., Suga, H., and Kageyama, K. (2021) Multiplex LAMP Detection of the Genus *Phytophthora* and Four *Phytophthora* Species *P. ramorum*, *P. lateralis*, *P. kernoviae*, and *P. nicotianae*, with a Plant Internal Control. *Microbes Environ ***36**: ME21019.

https://doi.org/10.1264/jsme2.ME21019

## Supplementary Material

Supplementary Material 1

Supplementary Material 2

Supplementary Material 3

## Figures and Tables

**Table 1.** Sequences of LAMP primer sets used in the present study.

LAMP primer set	Name		Sequence (5′-3′)	Length (bp)	Locus	Reference	Note
*Phytophthora ramorum*-specific	Pram	F3	TTACTGCACATGCTTTTATC	20	cytochrome *c* oxidase subunit I gene	This study	
		B3	GAATGTGCTTGTACACTTGA	20			
		FIP	AGGAAATGCCATATCTGGAGTGCCTGCTTTAATTGGTGGG	40			
		BIP	GGCTTTATTATTATTAGTTTTAAACTGTCCAACCAGTACC	40			
*Phytophthora lateralis*-specific	Plat	F3	TAATGATAGGTGCACCTGAT	20	cytochrome *c* oxidase subunit I gene	This study	
		B3	AATCTACTGAAGGTCCTGAA	20			
		FIP	CGATGAAACTAATAATAATATGGCTTTTCCACGTATGAA	39			
		BIP	GGCTATTGTAGAATCTGGTGGGGCTTGTACACTAGATAAC	40			
*Phytophthora kernoviae*-specific	Pker	F3	GGTAGTGTTGGTTTCGGC	18	rDNA-ITS region	This study	
		B3	CGTGCACACAAAAAATTGTTCC	22			
		FIP	TACACACTACTACGGTACACCTGCTTATTGTGTCTTTTTCCTTGC	45			
		BIP	ACTTTGTGTGCGAAGTAGAGAAAAGGTTTCGTCCCCTCGGC	41			
*Phytophthora nicotianae*-specific	Pnic	F3	CGGTGGAGGAGATGTCAGAT	20	rDNA-ITS region	Hieno *et al.* (2019)	
		B3	GTTCAGCCGAAGCCAACC	18			
		FIP	TCAGTTTAGTACATTTAAAATGAAGTGTCTTGCGATTGGT	40			
		BIP	AAGGCTGCTATTGTGGCAAAACACTCTTCCATTAACGCCG	40			
*Phytophthora* genus-specific	Physp	F3	TCTGCTTTTAACTAGATAGC	20	rDNA-ITS region	Hieno *et al.* (2020)	
		B3	CACCACTTTTCGAGCAAAGA	20			
		FIP	GCATACTTCCAGGACTAACCGTAATGCGAATTGCAGGA	38			
		BIP	CCTGTATCAGTGTCCGTACATCTCCTCCACCGACTACACG	40			
Plant	COX	F3	TATGGGAGCCGTTTTTGC	18	cytochrome *c* oxidase subunit I gene	This study (Tomlinson *et al.*, 2010 modified)	Original primer
		B3G	AACTGCTAAGGGCATTCC	18			Mix base in original B3 “R” replaced to “G”
		FIPA	ATGGATTTGACCTAAAGTTTCAGGGCAGGATTTCACTATTGGGT	44			Mix base in original FIP “R” replaced to “A”
		FIP2	ATGGATTTGACCTAAAGTTTCGGTCGCAGGATTTTACTTTCGGG	44			Additional FIP primer designed in this study
		BIPA	TGCATTTCTTAGGGCTTTCGGATCCAGCGTAAGCATCTG	39			Mix base in original BIP primer “R” replaced to “A”
		F-Loop	ATGTCCGACCAAAGATTTTACC	22			Original primer
		F-Loop2	ATGTTCGACCAGAGATTTTACC	22			Additional F-loop primer designed in this study
		B-Loop	GTATGCCACGTCGCATTCC	19			Original primer

**Table 2.** Detection of *Phytophthora* pathogens in inoculated plants by multiplex LAMP assays with an internal control.

Experiment	Host	Tissue	Inoculated *Phytophthora* species	Isolate*	Multiplex LAMP detection													
					*P. ramorum* with plant			*P. lateralis* with plant			*P. kernoviae* with plant			*P. nicotianae* with plant			Genus with plant	
					*P. ramorum*	Plant		*P. lateralis*	Plant		*P. kernoviae*	Plant		*P. nicotianae*	Plant		Genus	Plant
1	Rhododendron (*Rhododendron* sp.)	Detached leaf	*P. ramorum*	CBS 101553	+	–		–	+		–	+		NT	NT		+	–
		Detached leaf	*P. lateralis*	ATCC 201856	–	+		+	–		–	+		NT	NT		+	–
		Detached leaf	*P. kernoviae*	CBS 122049	–	+		–	+		+	–		NT	NT		+	–
		Detached leaf	None	–	–	+		–	+		–	+		NT	NT		–	+
	Japanese andromeda (*Pieris japonica* ssp. *japonica*)	Detached leaf	*P. ramorum*	CBS 101553	+	–		–	+		–	+		NT	NT		+	–
		Detached leaf	*P. lateralis*	ATCC 201856	–	+		+	–		–	+		NT	NT		+	–
		Detached leaf	*P. kernoviae*	CBS 122049	–	+		–	+		+	–		NT	NT		+	–
		Detached leaf	None	–	–	+		–	+		–	+		NT	NT		–	+
	Common camellia (*Camellia japonica*)	Detached leaf	*P. ramorum*	CBS 101553	+	–		–	+		–	+		NT	NT		+	–
		Detached leaf	*P. lateralis*	ATCC 201856	–	+		+	–		–	+		NT	NT		+	–
		Detached leaf	*P. kernoviae*	CBS 122049	–	+		–	+		+	–		NT	NT		+	–
		Detached leaf	None	–	–	+		–	+		–	+		NT	NT		–	+
2	Rhododendron (*Rhododendron* sp.)	Attached leaf	*P. ramorum*	ATCC MYA-2436	+	–		–	+		–	+		NT	NT		+	–
		Attached leaf	*P. ramorum*	CBS 101330	+	–		–	+		–	+		NT	NT		+	–
		Attached leaf	*P. lateralis*	CBS 168.42	–	+		+	–		–	+		NT	NT		+	–
		Attached leaf	*P. kernoviae*	CBS 122051	–	+		–	+		+	–		NT	NT		+	–
		Attached leaf	*P. kernoviae*	CBS 122208	–	+		–	+		+	–		NT	NT		+	–
		Attached leaf	*P. foliorum*	CBS 121655	–	+		–	+		–	+		NT	NT		+	–
		Attached leaf	*P. hibernalis*	CBS 522.77	–	+		–	+		–	+		NT	NT		+	–
		Attached leaf	None	–	–	+		–	+		–	+		NT	NT		–	+
	Japanese andromeda (*Pieris japonica* ssp. *japonica*)	Attached leaf	*P. ramorum*	ATCC MYA-2436	+	–		–	+		–	+		NT	NT		+	–
		Attached leaf	*P. ramorum*	CBS 101330	+	–		–	+		–	+		NT	NT		+	–
		Attached leaf	*P. lateralis*	CBS 168.42	–	+		+	–		–	+		NT	NT		+	–
		Attached leaf	*P. kernoviae*	CBS 122051	–	+		–	+		+	–		NT	NT		+	–
		Attached leaf	*P. kernoviae*	CBS 122208	–	+		–	+		+	–		NT	NT		+	–
		Attached leaf	*P. foliorum*	CBS 121655	–	+		–	+		–	+		NT	NT		+	–
		Attached leaf	*P. hibernalis*	CBS 522.77	–	+		–	+		–	+		NT	NT		+	–
		Attached leaf	None	–	–	+		–	+		–	+		NT	NT		–	+
3	Tomato (*Solanum lycopersicum*)	Fruit	*P. capsici*	CH01CUCU10	NT	NT		NT	NT		NT	NT		–	+		+	–
		Fruit	*P. nicotianae*	GK10Eg1	NT	NT		NT	NT		NT	NT		+	–		+	–
		Fruit	None	–	NT	NT		NT	NT		NT	NT		–	+		–	+
	Eggplant (*Solanum melongena*)	Fruit	*P. capsici*	CH01CUCU10	NT	NT		NT	NT		NT	NT		–	+		+	–
		Fruit	*P. nicotianae*	GK10Eg1	NT	NT		NT	NT		NT	NT		+	–		+	–
		Fruit	None	–	NT	NT		NT	NT		NT	NT		–	+		–	+

“+”; detected, “–”; not detected, NT; not tested. *CBS: CBS-KNAW collection, Westerdijk Fungal Biodiversity Institute, the Netherlands. ATCC: American Type Culture Collection, the United States.
