# Supplementary material for: Multiplex LAMP Detection of the Genus Phytophthora and Four Phytophthora Species P. ramorum, P. lateralis, P. kernoviae, and P. nicotianae, with a Plant Internal Control
Source: Microbes Environ. 2021 Jun 10;36(2):ME21019. doi: 10.1264/jsme2.ME21019 (PMC8209452; doi:10.1264/jsme2.ME21019)
Supplement: Supplementary file 1 — Supplementary Material 1 [file 36_21019_s1.pdf]

Table S2 Sequences and accession numbers of cytochrome *c* oxidase subunit I gene used for LAMP primer design (see Fig. S4).

Family	Scientific name	Common name	Accession numbers	Reference
**Woody plant**				
Adoxaceae	*Sambucus canadensis*	American black elderberry	AF193965	Parkinson et al., *Current Biology* , 1999
Calycanthaceae	*Calycanthus floridus*	Eastern sweetshrub	AF193955	Parkinson et al., *Current Biology* , 1999
Lauraceae	*Laurus nobilis*	Bay laurel	AF193956	Parkinson et al., *Current Biology* , 1999
Magnoliaceae	*Magnolia grandiflora*	Southern magnolia	AF020568	Bowe et al., *PNAS* , 2000
Magnoliaceae	*Liriodendron tulipifera*	Tulip tree	AF193959	Parkinson et al., *Current Biology* , 1999
Proteaceae	*Grevillea robusta*	Southern silky oak	AF193961	Parkinson et al., *Current Biology* , 1999
Araucariaceae	*Agathis australis*	Kauri	AF020557	Bowe et al., *PNAS* , 2000
Cupressaceae	*Chamaecyparis pisifera*	Sawara cypress	EF053140	Nian et al., *Chinese Sci Bull* , 2009
Cycadaceae	*Cycas revoluta*	Sago palm	AF020562	Bowe et al., *PNAS* , 2000
Ginkgoaceae	*Ginkgo biloba*	Ginkgo	AF020565	Bowe et al., *PNAS* , 2000
Pinaceae	*Pinus strobus*	Eastern white pine	AF020574	Bowe et al., *PNAS* , 2000
Pinaceae	*Tsuga canadensis*	Eastern hemlock	AF020582	Bowe et al., *PNAS* , 2000
Sciadopityaceae	*Sciadopitys verticillata*	Japanese umbrella-pine	AF020576	Bowe et al., *PNAS* , 2000
Taxaceae	*Taxus baccata*	European yew	AF020579	Bowe et al., *PNAS* , 2000
Taxaceae	*Torreya nucifera*	Japanese torreya	AF020580	Bowe et al., *PNAS* , 2000
**Herbaceous plant**				
Amaranthaceae	*Beta vulgaris*	Common beet	M57645	Harada et al., *Proc. Sugar Beet Res. Assoc. Jpn.* , 1987
Fabaceae	*Glycine max*	Soybean	M16884	Grabau, *Plant Mol. Biol.* , 1986
Plantaginaceae	*Digitalis purpurea*	Foxglove	AJ223415	Cho et al., *PNAS* , 1998
Poaceae	*Triticum aestivum*	Common wheat	Y00417	Bonen et al., *NAR* , 1987

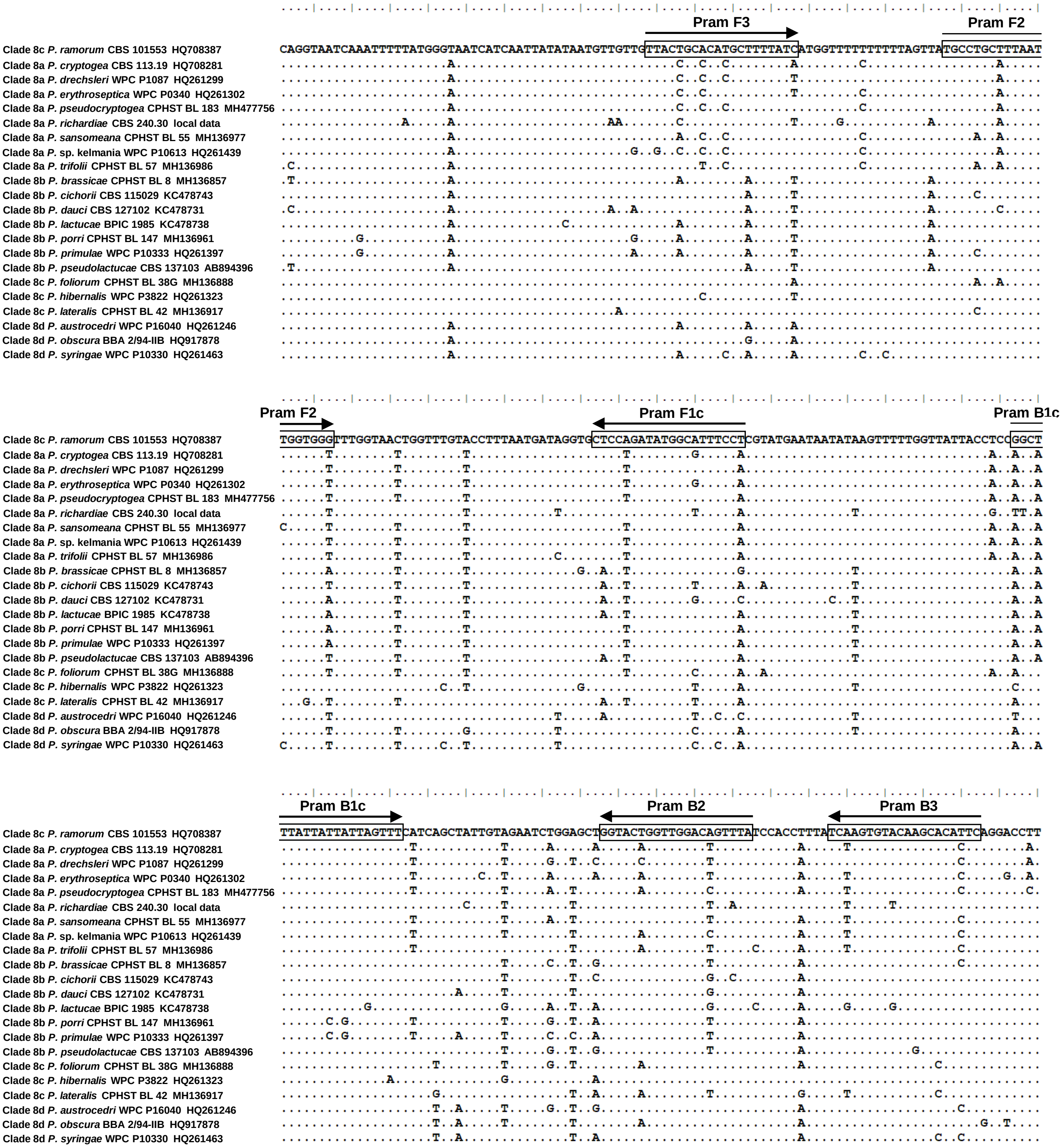

Fig. S1 Design of the *Phytophthora ramorum* species-specific LAMP primer set (Pram) based on sequences of the cytochrome *c* oxidase subunit I (*cox*1) gene. A nucleotide sequence alignment of a partial *cox*1 gene from *P. ramorum* and closely related species in clade 8 (Abad et al., 2019) was used to design the species-specific primer set. Nucleotides shared with the *P. ramorum* sequence are shown as dots (.). The boxed regions show the sequences of the LAMP primers F3, B3, FIP (F1c to F2), and BIP (B1c to B2). Arrows indicate the 5' to 3' direction. The accession number for each sequence is shown after each isolate name. CBS: CBS-KNAW collection, Westerdijk Fungal Biodiversity Institute the Netherland. ATCC: American Type Culture Collection the United States. WPC: World Phytophthora Genetic Resource Collection the United States. CPHST BL: USDA-APHIS-Center for Plant Health Science and Technology Beltsville Laboratory the United States. BBA: Biologische Bundesanstalt für Landund Forstwirtschaft Germany. BPIC: Benaki Phytopathological Institute Culture Collection Greece. Accession numbers are cited from the NCBI nucleotide database.

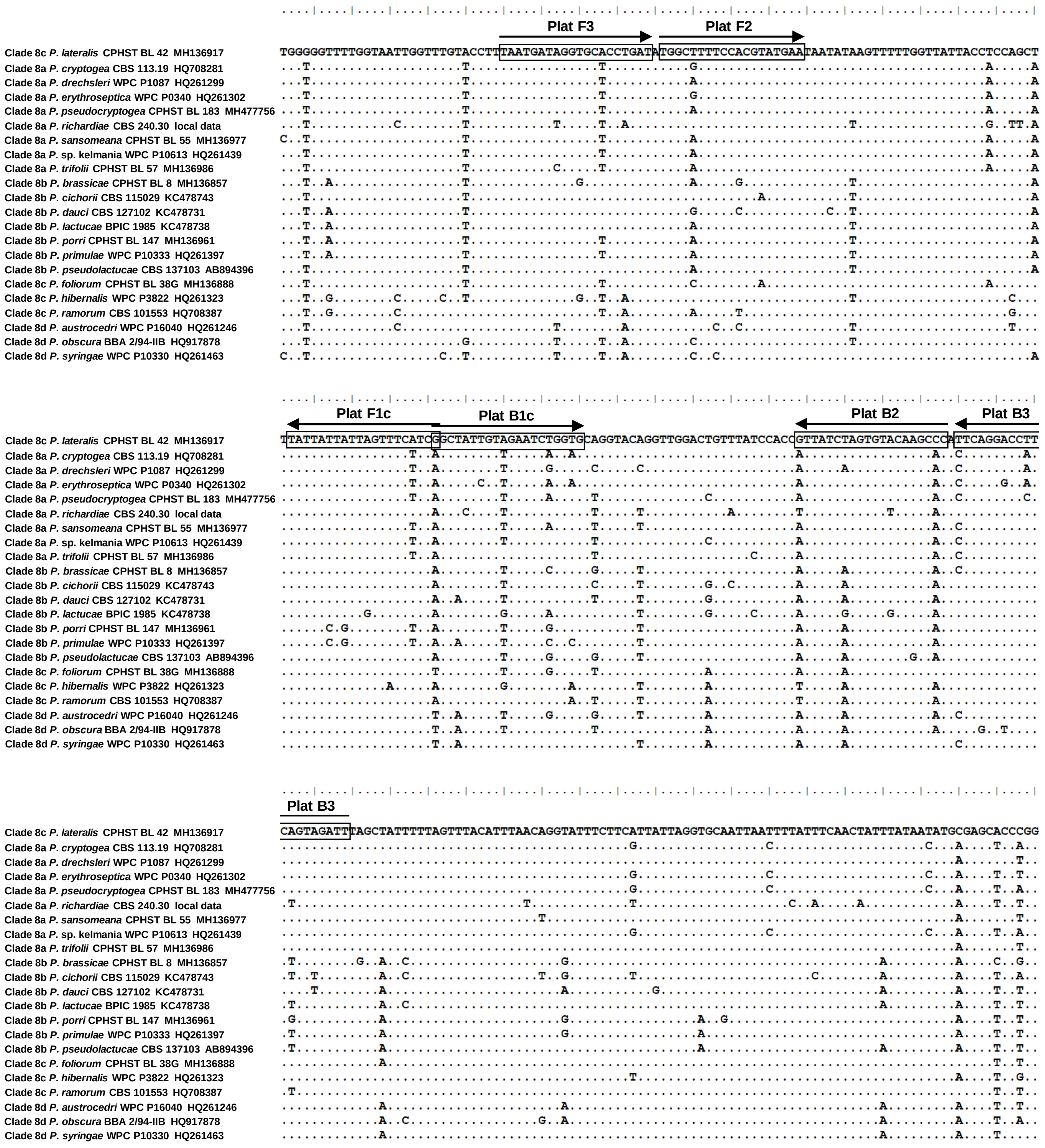

Fig. S2 Design of the *Phytophthora lateralis* species-specific LAMP primer set (Plat) based on the cytochrome *c* oxidase subunit I (*cox*1) gene. The primers were designed using *cox*1 gene sequence of *P. lateralis* and those closely related species, as shown in Fig. S1. The culture collections of isolates are listed in the Fig. S1 legend.

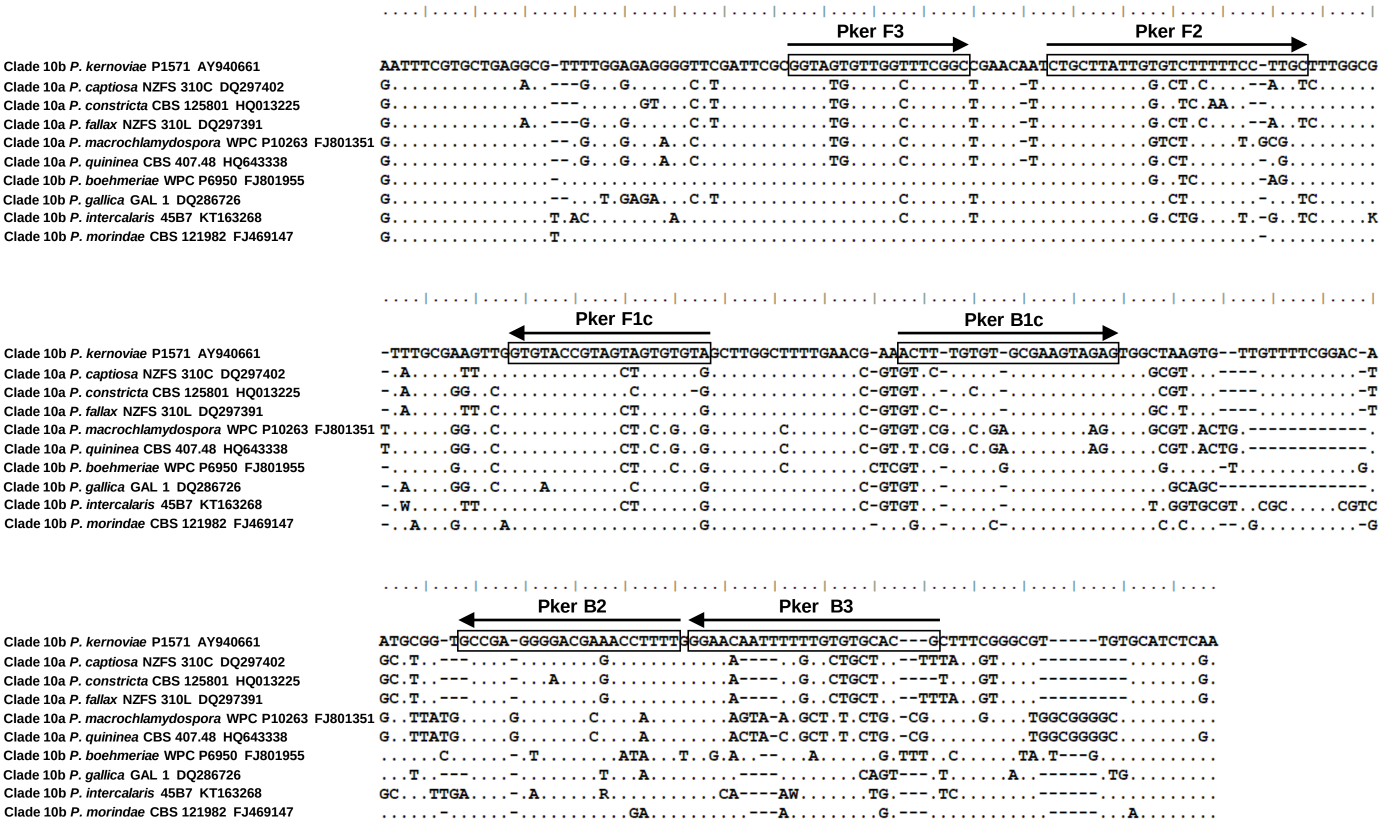

Fig. S3 Design of the *Phytophthora kernoviae* species-specific LAMP primer set (Pker) based on rDNA-ITS sequences. The primers were designed using rDNA-ITS sequence of *P. kernoviae* and those closely related species in clade 10 (Abad et al., 2019). The culture collections of isolates are listed in the Fig. S1 legend. NZFS: New Zealand Forest Research Institute Culture Collection New Zealand.

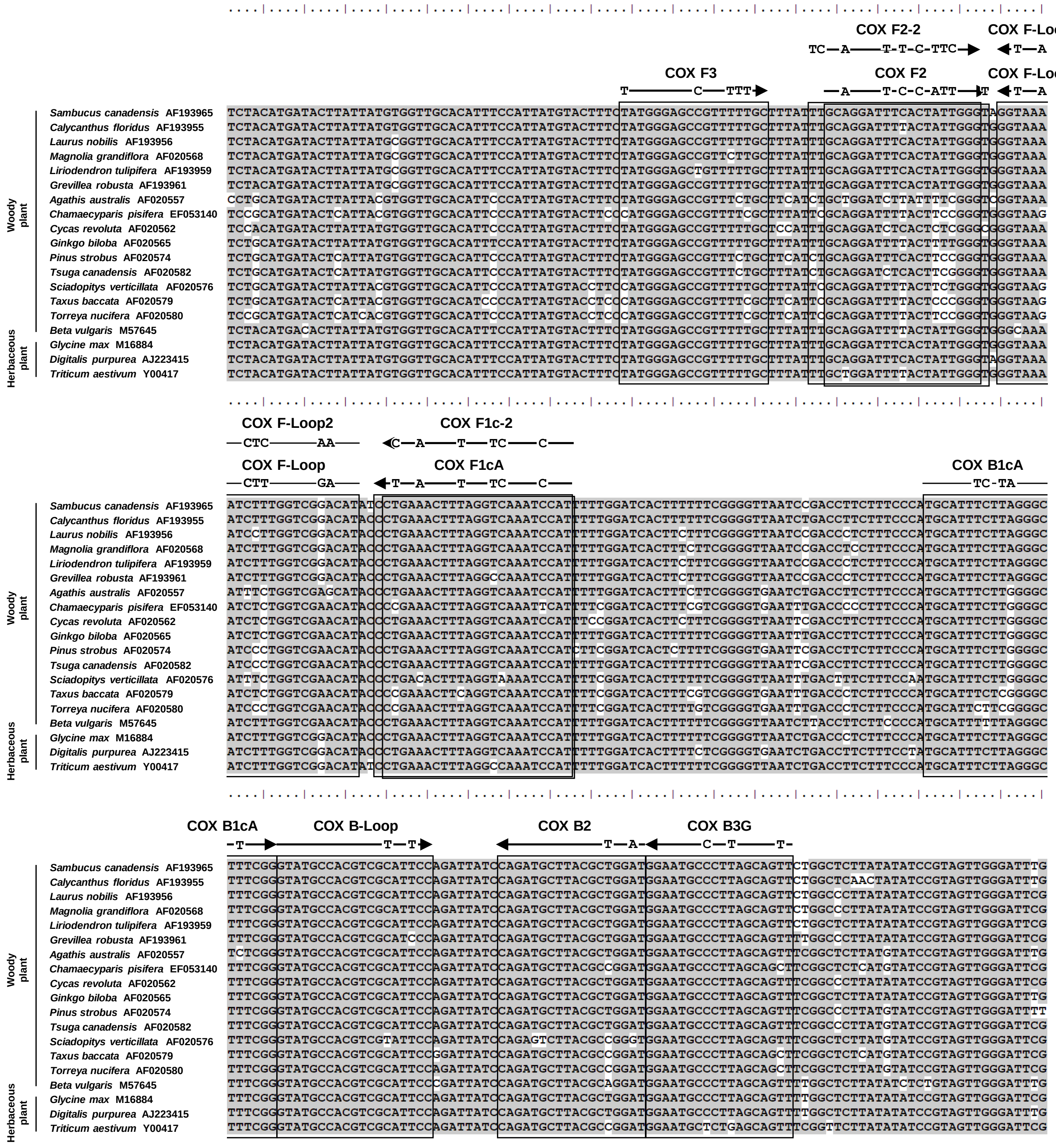

Fig. S4 Design of the modified plant LAMP primer set based on the cytochrome *c* oxidase subunit I (*cox*1) gene. A nucleotide sequence alignment of a partial *cox*1 gene from plant species (16 woody plants and 3 herbaceous plants) to design the plant LAMP primer set. Identical and similar nucleotides are shown with gray and white backgrounds, respectively. The boxed regions show the sequences of the LAMP primers F3, B3G, FIPA (F1cA to F2), FIP2 (F1c-2 to F2-2), BIPA (B1cA to B2), F-Loop, F-Loop2, and B-Loop. Arrows indicate the 5’ to 3’ direction. Nucleotides on the arrows indicate the nucleotides used for the primer design, at sites that were not identical in all plants. The accession numbers for the sequences are shown after each species. See Table S2 for details.

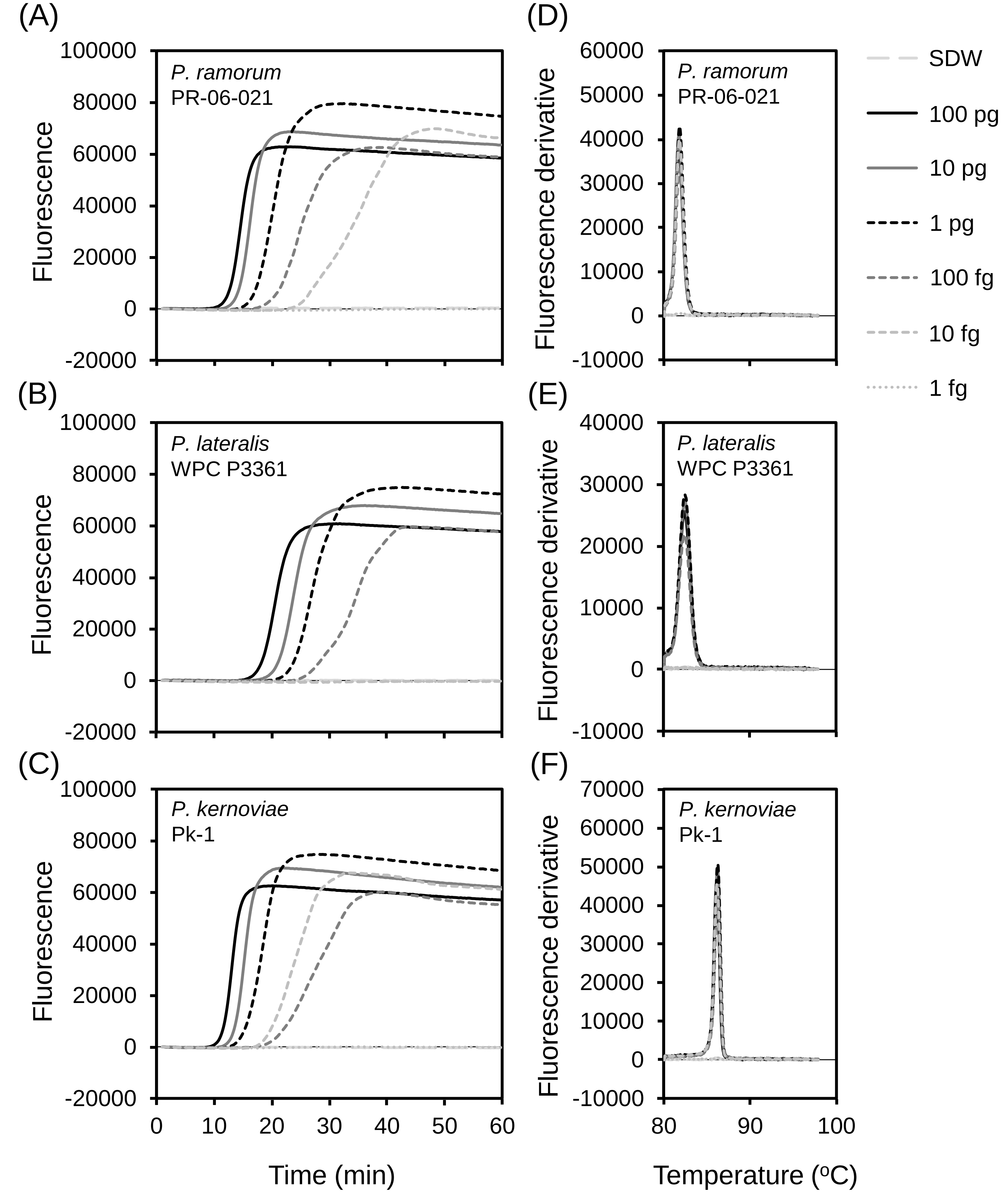

Fig. S5 Sensitivity of the *Phytophthora* species-specific LAMP assays. Mycelial DNA extracted from *P. ramorum* PR-06-021 (A and D), *P. lateralis* WPC P3361 (from the World Phytophthora Genetic Resource Collection), (B and E), and *P. kernoviae* Pk-1 (C and F) was serially diluted (1 fg to 100 pg per reaction) and subjected to LAMP assays with the corresponding species-specific primer sets. (A–C) Amplification curves. (D–F) After amplification at 65°C for 60 min, the fluorescence derivative data during the annealing phase (98°C to 80°C) were obtained. SDW: sterilized deionized water.
